# Discrimination and Proper Use of Polygoni Multiflori Radix, Cynanchi Wilfordii Radix, and Cynanchi Auriculati Radix in Korea: A Descriptive Review

**DOI:** 10.1155/2015/827380

**Published:** 2015-10-11

**Authors:** Beom-Joon Lee, Kyungjin Lee

**Affiliations:** College of Korean Medicine, Kyung Hee University, 26 Kyungheedae-ro, Dongdaemun-gu, Seoul 130-701, Republic of Korea

## Abstract

Polygoni Multiflori Radix (PMR), Cynanchi Wilfordii Radix (CWR), and Cynanchi Auriculati Radix (CAR) are very popular herbal medicines in Traditional Korean Medicine, Traditional Chinese Medicine, and Kampo Medicine. However, the plant origins, efficacies, and traditional uses of these herbal medicines differ. In Korea, PMR is called *Ha Su O* (*He Shou Wu* in China), and CWR is called *Baek Ha Su O* or *Baek Su O* (*Bai Shou Wu* in China). *Baek Su O* refers to CWR in Korea and CAR in China. CAR has not been used as a traditional herbal medicine, and it cannot be legally used as a food or food ingredient in Korea. However, CAR is cultivated in Korea and imported from China. Because the morphology of CWR and CAR is very similar, they are often confused and misused in Korea. This review discusses the reasons for the confusion and misuse of these substances in Korea and provides the exact plant origins, efficacies, uses, components, and toxicities of PMR, CWR, and CAR so that they can be correctly understood and used.

## 1. Introduction

In Korea, the Ministry of Food and Drug Safety (MFDS) permits the use of 189 medicinal herbs, including Ginseng Radix, Angelicae Gigantis Radix, Polygoni Multiflori Radix (PMR), and Cynanchi Wilfordii Radix (CWR), for use in foods and as traditional herbal medicines [1]. However, various minor and major problems have arisen relating to their use owing to a lack of correct understanding of these medicinal herbs.

Traditional medicines have long been used in Korea, China, and Japan. Traditional Korean Medicine, Traditional Chinese Medicine (TCM), and Kampo Medicine have a number of similarities and differences due to their geographical, cultural, political, and climatic connections. In particular, there are many differences in their use of herbal medicines. PMR and CWR are two classic examples.

In 2015 in Korea, information regarding foods and functional health foods that contain CWR caused great social confusion and resulted in an economic loss. In recent years, functional health foods containing CWR have been popularly used for preventing or treating climacteric syndrome in women, as the MFDS permits complex extracts containing CWR as a functional ingredient for this purpose. These functional health foods created a revenue of more than $100 million in 2014. However, 301 cases of side effects from functional health foods containing CWR were reported by MDFS, which accounted for 17% of total reported side effects cases (1,733 cases) induced by functional health foods [2]. Furthermore, the MFDS reported that most of these functional foods contained Cynanchi Auriculati Radix (CAR) with CWR, and in some instances only CAR was present in place of CWR [2].

By law, CAR cannot be used as food or as a food ingredient in Korea because there is no evidence to support its use, and, in China, it has been reported to have toxicities. Therefore, people who consumed foods or functional foods containing CWR were subject to great confusion and this resulted in the mass return of such products and subsequent refunds.

The MFDS collected samples of all CWR-related products, which are produced in Korea, and discarded all the products containing CAR after DNA analysis. In addition, in order to relieve the anxiety of the people, the MFDS publicly announced the names of companies and products that did not contain CAR, as well as the products for which presence of CWR could not be determined.

The primary cause of these events was the misunderstanding of PMR, CWR, and CAR and these three medicinal herbs often have been sold as* Ha Su O* or* Baek Su O* without distinction in the market.

PMR, which has long been used as a traditional herbal medicine, is called* Ha Su O* in Korea (*He Shou Wu* in China). However, there is some confusion of the use of this herbal medicine because of misunderstandings of the PMR name. In Korea, PMR is often separated into red PMR (*Jeok Ha Su O* in Korea) and white PMR (*Baek Ha Su O* or* Baek Su O* in Korea). Red PMR is a root tuber of* Polygonum multiflorum* (PM) Thunberg, while white PMR is the root tuber of* Cynanchum wilfordii* (CW) Hemsley [3–5]. Similarly, the plant origins of red PMR and white PMR are completely different, even though the Korean names are similar. Therefore, CWR is commonly misused as PMR in Korea. In addition, CAR is commonly misused as CWR or PMR because of its very similar external morphology to CWR. Furthermore, the plant origin of* Baek Su O* (*Bai Shou Wu* in China) differs between Korea and China. CWR is* Baek Su O* in Korea [4], while CAR is* Baek Su O* in China [6].

The plant origins, efficacies, and traditional uses of these herbal medicines differ from each other. Therefore, people should use these medicinal herbs appropriately after correctly understanding the differences between them. In the current review, we provide a comprehensive overview of PMR, CWR, and CAR, including their exact plant origins, efficacies, uses, components, and toxicities so that they can be correctly understood and used.

## 2. Summary of Terms

Chinese characters have long been used in the Korean language. Their meaning is the same, but their pronunciation is different. In the current review, the Korean names of the literature, herbal medicines, and prescriptions are written in English according to their Korean pronunciation, and the Chinese names of the literature and herbal medicines are written in English according to the Chinese pronunciation.* Ha Su O* is* He Shou Wu* in Chinese, and* Baek Su O* is* Bai Shou Wu* in Chinese. Other terms are written in English according to the international standard of the World Health Organization of traditional medicine terms in the western pacific region [7].

## 3. Origins

The root tuber of PM has been used for centuries in Korea, China, and Japan as the traditional medicine of PMR [5, 8, 9]. PMR was first mentioned as* He Shou Wu* in* Ri Hua Zi Ben Cao*, which is an ancient Chinese text that was written during the Wu Ddai Shi Guo [3, 6]. In Korea, PMR was first recorded as* Ha Su O* in* Dong Ui Bo Gam*, which is an ancient Korean text that was written during the Cho Sun dynasty. In addition, PMR was called* On Jo Rong* in the Gangwon province and* Sae Park Bul Hui* in the Hwanghae province [10]. Today, PM is registered as the plant origin of PMR in the Korean, Chinese, and Japanese pharmacopoeia [5, 8, 9].

The root tuber of CW has been used as CWR, which is a traditional medicine, in Korea [4]. CWR was first recorded as* Baek Ha Su O* in the traditional Korean texts* Bang Yak Hap Pyeon* (1884) and* Dong Ui Su Se Bo Won* (1894) [3]. CWR is recorded as* Baek Su O* in the Korean Herbal Pharmacopoeia [4]. Therefore, CWR is called* Baek Ha Su O* or* Baek Su O* in Korea. However, no traditional medical literature refers to CWR as* Baek Su O* in Korea. Confusingly, a herbal medicine that is called* Baek Su O* was first mentioned in* Shan Dong Zhong Yao* (1959) in China [3, 6]. The herbal plants that are currently called* Baek Su O* in Korea and China are different. The origin of the Korean* Baek Su O* is a root tuber of CW [4], while the origin of the Chinese* Baek Su O* is a root tuber of* Cynanchum auriculatum* (CA) Royle ex Wight and* Cynanchum bungei* Decne [6]. CAR, which is the Chinese* Baek Su O*, is called* Yi Yeop Wu Pi So*, and it is not registered in the Korean Pharmacopoeia and Korean Herbal Pharmacopoeia. CAR has not been used as a traditional medicine and food in Korea. Therefore, the MFDS of Korea prohibits the use of CAR as a food or food ingredient. In contrast, CAR is permitted for use as a drug or food in China. CWR, which is the Korean* Baek Su O*, is called* Ge Shan Xiao* in China, and it is not used nearly as much as a drug or food in China.

## 4. Morphological Botany

**Figure 1:**
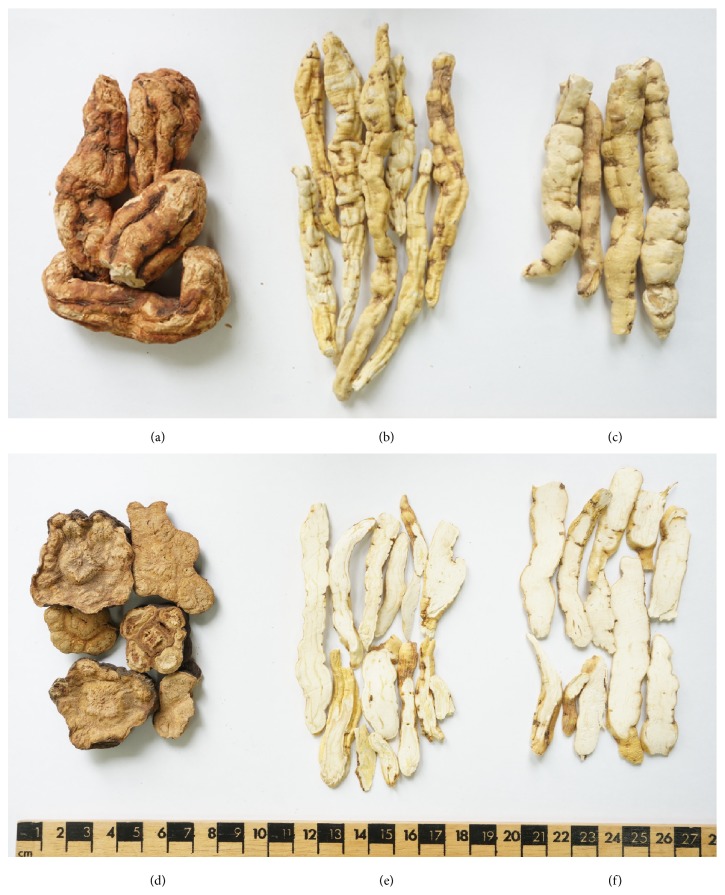
The roots (one year old) of (a)* Polygonum multiflorum* (PM) Thunberg, (b)* Cynanchum wilfordii* (CW) Hemsley, and (c)* Cynanchum auriculatum* (CA) Royle ex Wight. Decocting pieces of (d) PM, (e) CW, and (f) CA.

PM, CW, and CA are all climbing plants. PM is an herbaceous plant that belongs to the Polygonaceae family. Its stem twines to the right, and its leaves alternate. It does not contain a white milky liquid, and its roots are fusiform and reddish-brown like a sweet potato [11] (Figure 1(a)). CW and CA are herbaceous plants that belong to the Asclepiadaceae family. Their stems twine to the left, their leaves are opposite each other and contain a white milky liquid, and their roots are thickened and yellowish brown [11]. Therefore, CW and CA are easily distinguished from PM by these morphological characteristics.

The morphological characteristics of CW and CA are mostly similar (Figure 1). However, they can be distinguished by their stipule and flower color. A stipule is present in CW and absent in CA. The flower color of CW is yellow-green, while it is yellowish white in CA [11]. However, the external and internal morphology of CWR and CAR are very similar, except that CAR is generally longer and thicker than CWR is (Figure 1). Therefore, it is not easy to distinguish the roots of CW and CA, and it is almost impossible to distinguish CWR and CAR in the mixture.

## 5. Pharmacology

PMR has been used to treat sores and abscesses, scrofula, rubella, deficiencies, constipation due to intestinal dryness from the effects of detoxification, sallow complexions due to blood deficiency, dizziness, tinnitus, premature graying of the hair and beard, weakness and pain in the waist and knees, paralysis, flooding and spotting, vaginal discharge, and hypercholesterolemia with tonifying of the liver and kidney; disperse abscesses; interrupt malaria; moisten the intestine; relax the bowels; nourish the essence and blood; blacken the beard and hair; and strengthen the sinew and bone after preparation with black bean juice in Korea and China [8, 10]. PMR has been reported to have various pharmacological activities, including acetylcholinesterase inhibition, neuroprotection, antioxidation, immunomodulation, antihyperlipidemia, hepatoprotection, anticancer effects, and anti-inflammation [12].

CAR has been used to treat weakness and pain in the waist and knee, impotence, spermatorrhea, dizziness, tinnitus, palpitations, insomnia, loss of appetite, infantile malnutrition with accumulation, postpartum milk decreases, sores and abscesses, and snake bites with tonifying of the liver and kidney; strengthen the sinew and bone; nourish the essence and blood; fortify the spleen; and promote digestion, and it has been used for detoxification in TCM [6]. It has been reported to have a number of effects, including antioxidant, immunomodulating, antitumor, antihyperlipidemia, hair growth promoting [6], and antidepressant effects [13]. However, to the best of our knowledge, there are no descriptions of the prescribing of CAR as a medicine or food in Korea in the ancient literature or other references.

References to some prescriptions that contain CWR without descriptions of the characteristics of CWR, including its flavor, medicinal nature, or efficacy, appear in the ancient writing of* Bang Yak Hap Pyeon* (1884) in Traditional Korean Medicine. The list of prescriptions and their indications was as follows:* Oryeong-tang* for wind cold dampness impediment and tetanus;* Boanmanryeong-dan* for hemiparalysis;* Seungyangikgi-tang* for qi deficiency in Greater Yang syndrome in a Lesser Yin person;* Ogan-tang* for prolapse of the uterus;* Muki-hwan* for deficiency syndrome;* Gabil-hwan* for food accumulation, aggregation accumulation, vomiting, diarrhea, and cholera;* Paeo-tang* for static blood;* Homa-san* for wind-heat urticaria; and* Kyeoleum-dan* for refractory bloody stool. Another list of prescriptions that contain CWR was in the* Dong Ui Su Se Bo Won* (1894), and it included* Hyangsayukgunja-tang*,* Seungyangikgi-tang*,* Hwanggigyeji-tang*,* Palmulgunja-tang*,* Hyangbujapalmul-tang*,* Jeokbaekhaogwanjung-tang*,* Sanmil-tang*, and* Baekhaobujalijung-tang* for treating the syndromes of a Lesser Yin person. However, CWR is an herbal medicine that is known for treating dizziness, vertigo, insomnia, forgetfulness, early white beard and hair, impotence, spermatorrhea, weakness of the waist and knee, an absence of transport of spleen deficiency, abdominal distension, loss of appetite, diarrhea, postpartum milk lessening, and mouth sores with the effects of tonifying the liver and kidney; strengthening the sinew and bone; fortifying the spleen; and causing detoxification in TCM [6]. Recently, anti-inflammatory effects [14], antihyperlipidemia effects [15–17], and antihypertensive effects [18] of CWR have been elucidated.

## 6. Phytochemistry

The components of PMR are the following: resveratrol; polydatin; rhaponticoside; polygonumosides A, B, C, and D; emodin; chrysophanol; physcion; rhein; emodin-1,6-dimethyl ether; citreorosein; fallacinol; 2-acetylemodin; tricin; rutin; luteolin; quercetin; kaempferol; isoorientin; apigenin; hyperoside; vitexin; quercetin-3-O-arabinoside; polygonflavanol A; phosphatidylethanolamine; copaene; eicosane; hexanoic acid; hexadecanoic acid ethyl ester; squalene; catechin; epicatechin; 3-O-galloyl-procyanidin B2; gallic acid; methyl gallate; daucosterol; *β*-sitosterol; and schizandrin [12].

The components of CWR are as follows: cynandione A [19], cynanoneside B, p-hydroxyacetophenone, 2,5-dihydroxyacetophenone, 2,4-dihydroxyacetophenone, wilfoside K1N, wilfoside C1N [20], *β*-sitosterol, wilfoside C3N, methyleugenol, wilfoside C1G, cynauriculoside A, daucosterol, acetovanillone, sucrose, geniposide, succinic acid, bungeiside A [21], and cynanchone A [22].

The components of CAR are the following: cynandione A [19]; cynanoneside B; p-hydroxyacetophenone; 2,5-dihydroxyacetophenone; 2,4-dihydroxyacetophenone; wilfoside K1N; wilfoside C1N [20]; C21-steroidal glycoside [23]; cyanoauriculosides F, G, and H [24]; wilfoside C3N [25]; taraxasterol acetate; cynanchone A; succinic acid; betulinic acid; kidjoranin [25]; auriculoside A [26]; caudatin; metaplexigenin; azelaic acid; wilforibiose; sucrose; 1-O-hexadecanolenin; beta-amyrin acetate; acetylquinol; beta-sitosterol; and daucosterol [27].

## 7. Toxicology

PMR or prescriptions that include PMR have been reported as the cause of 450 cases of liver toxicity, including jaundice, fatigue, anorexia, or yellow or tawny urine, in 76 articles [28]. In addition, a 50% alcohol extract of PMR induced liver injury in a lipopolysaccharide-based idiosyncratic hepatotoxicity rat model [29]. The ethanol extract and water extract of PMR both show hepatotoxicity, while the hepatotoxicity from the ethanol extract is much stronger [30]. The oral administration of the water extract of raw PMR is much more toxic than the acetone extract in Kunming mice. In addition, the oral administration of the acetone extract of raw PMR is much more toxic than that of the acetone extract of processed PMR [31]. In addition to these reports, many studies have described the hepatotoxicity of PMR in China [32–34] and Korea [35–38]. Most of these reports were related to individuals who were taking PMR without a doctor's prescription or for a long time or those who overdosed. PMR is known in traditional medicine to have slight poisonous effects. Therefore, it is usually used after it is prepared with black bean juice that reduces its toxicity and increases its efficacy [8, 39]. Recently, Wu et al. reported that the toxicity of PMR is decreased after this preparation [31]. Therefore, the intake of PMR as an herbal medicine or food without preparation or a doctor's prescription has great potential for causing liver toxicity.

CWR has been reported to be toxic in rats and mice. The intraperitoneal administration of the 70% ethanol extract at doses of 50, 100, 200, and 300 mg/kg did not show toxicity, but doses of 500 (10%) and 1,000 mg/kg (20%) caused death [40]. In a subchronic toxicity study, the 4-week oral administration of the 70% ethanol extract of PMR in rats resulted in death in groups treated with 300, 500, and 1,000 mg/kg during the experimental period, and the toxicities were highly dose dependent [40].

CAR has also been reported to be toxic and cause increased saliva, vomiting, spasm, difficulty in breathing, and slowing of the heart beat in the China Plant Collection Database [41]. In addition, most of the* Cynanchum* family (Asclepiadaceae) is toxic, and the toxicities of the root and white milky liquid are greater than those of the other parts [41]. Lu et al. reported that CAR cannot be used as a food because the intake limit for humans (bodyweight, 60 kg) was inferred to be 2.4 g/day from the intake of rats of a rodent diet that contained raw CAR and that caused severe weight loss and death [42]. Although this experiment had a number of problems and the quantity of CAR was higher in the diet, the rats did not eat much, and it still caused death. These results are important because the rats died, and a higher dose of CAR would have caused much more death. In addition, CAR is listed as a toxic plant in the Food and Drug Administration's Poisonous Plant Database in the US with reference to abortion activity in sows [43]. Therefore, the intake of large amounts of CAR should be avoided in China [6].

## 8. Future Perspectives and Conclusions

In Korea, PMR, CWR, and CAR are used as traditional herbal medicines, foods, or food ingredients. However, these substances can be confused and misused due to misunderstandings of these medicines. The plant origins, components, efficacies, potential applications, and toxicities of these medicines differ from each other. In addition, CWR is sometimes misused as PMR because of the similar Korean names of* Baek Ha Su O* (*Baek Su O*) and* Ha Su O*, respectively. CAR is also misused as CWR because of their similar morphology and its cheap price. CAR is even misused as PMR. All cases of imported* Ha Su O* from China are all CAR because* Baek Su O* is CAR in China. However, CWR is only referred to as* Baek Su O* in Korea. Therefore, the MFDS of Korea prohibits the use of CWR or CAR as PMR [5] and the use of CAR as CWR. In addition, no CA parts, including the root, can legally be used as food or food ingredients because its safety as a food has not been proved in Korea [44]. However, it can be used as an herbal medicine by Korean Medicine doctors.

PM and CW are widely distributed and have long been cultivated in Korea, but CA was distributed and cultivated only in China. CA seeds were first imported from China and cultivated since the 1990s in Youngju city in Kyungsangbuk-do province [45]. The external morphology of CAR is very similar to that of CWR. The growth rate of CAR is faster than that of CWR, and CA is much more resistant to blight and harmful insects. Therefore, farmers prefer to cultivate CAR over CWR. However, CAR cannot be used as a food or food ingredient, and it is not used as an herbal medicine in Korea. Thus, CAR is disguised as CWR or PMR or mixed with CWR. In addition, according to a presentation by the Ministry of Agriculture, Food and Rural Affairs, 187 tons of* Baek Su O* (CWR) was produced in Korea and 79 tons of* Baek Su O* was imported from China in 2014. However, Chinese* Baek Su O* is not CWR but CAR, and Chinese* Baek Su O* (CAR) is being sold as CWR or even as PMR in local markets.

Therefore, an exact understanding of these herbal medicines and their methods of distinction is needed for the correct use of these substances as a food or drug. However, it is difficult to distinguish between CWR and CAR because their external morphology and components are very similar. Li et al. suggested that conduritol F might be a unique marker compound that can be used for discriminating between CWR and CAR because it exists only in CWR [20]. CWR can therefore be distinguished from CAR by a high-performance liquid chromatography analysis of conduritol F, but it is impossible to identify CAR in the mixtures or products that contain both CWR and CAR. A DNA analysis can then be used to identify CAR in mixtures or products in Korea. However, this method is greatly limited because most mixtures or products are extracts after boiling. Thus, there is no way to identify CAR in the products that contain CWR and CAR after heating. For this reason, although the MFDS collected a total of 207 CWR-related products and conducted DNA analyses, they could not confirm whether CAR was present in 157 products. They further found that 40 products contained CAR and only 10 products did not contain CAR. As a result, the MFDS discarded all 40 products that contained CAR and allowed manufacturers to sell the other 157 products after proving that they did not contain CAR [2]. Therefore, phytochemical studies need to be conducted in order to find an index component that exists only in CAR.

PMR, CWR, and CAR all have some toxicity, which has been described in several case reports. PMR is used as an herbal medicine by doctors of traditional medicine after it is prepared with black bean juice, which reduces the toxicity and increases its efficacy in traditional medicine. CAR and CWR are also carefully used in clinics. Most of their toxicities were induced by their long-term use, overdose, or individuals taking it without a doctor's prescription. Case reports on their toxicities and side effects are useful for determining the safe use of herbal medicines. In Korea, many cases have been reported on the hepatotoxicity of PMR. Most of these toxic cases were also caused by individuals taking the drug without a doctor's prescription. However, most people consider CWR to be PMR. In addition, most people cannot distinguish between CWR and CAR and do not know the exact amount they had taken. Thus, these reports cannot be used as scientific proof of the safe use of herbal medicine. It is important that people understand the characteristics of PMR, CWR, and CAR and abstain from using these medicines on their own without a doctor's guidance. Careful toxicological studies of these medicines are needed in order to determine safety guidelines.

In conclusion, PMR and CWR are very popular traditional herbal medicines and food ingredients in Korea, and CAR is a popular traditional herbal medicine in China. Their plant origins, efficacies, uses, components, and toxicities differ from each other. However, they are often confused and misused because of the confusion in their drug names and their similar morphologies. Therefore, people should try to understand their characteristics and be able to distinguish between them. In addition, it is necessary to rename the drug names of CWR and CAR in Korea because of the confusion and misuse that are caused by the same appellative name being given to CAR and CWR. The name* Baek Su O* does not appear in traditional Korean literature but does appear in traditional Chinese literature. CWR was called* Baek Ha Su O* in traditional Korean literature. Therefore, the drug name of CWR should be changed to* Baek Ha Su O*, and the drug name of CAR should be changed to* Baek Su O* in Korea in order to prevent these misunderstandings and misuses. Further, more comparative studies of their efficacy, phytochemistry, and toxicity are needed. In particular, more phytochemical studies are needed to find CAR in boiled herbal mixtures, along with further scientific and clinical research of the toxicology.
